# Aging and serum MCP-1 are associated with gut microbiome composition in a murine model

**DOI:** 10.7717/peerj.1854

**Published:** 2016-03-31

**Authors:** Melissa N. Conley, Carmen P. Wong, Kyle M. Duyck, Norman Hord, Emily Ho, Thomas J. Sharpton

**Affiliations:** 1 School of Biological and Population Health Sciences, Oregon State University, Corvallis, OR, United States; 2 Center for Health Aging Research, Oregon State University, Corvallis, OR, United States; 3 Department of Microbiology, Oregon State University, Corvallis, OR, United States; 4 Linus Pauling Institute, Oregon State University, Corvallis, OR, United States; 5 Moore Family Center for Whole Grain Foods, Nutrition and Preventive Health, Oregon State University, Corvallis, OR, United States; 6 Department of Statistics, Oregon State University, Corvallis, OR, United States

**Keywords:** Aging, Inflammation, Inflammaging, Microbiome, Immunity, Immunosenescence, Mice

## Abstract

**Introduction.** Age is the primary risk factor for major human chronic diseases, including cardiovascular disorders, cancer, type 2 diabetes, and neurodegenerative diseases. Chronic, low-grade, systemic inflammation is associated with aging and the progression of immunosenescence. Immunosenescence may play an important role in the development of age-related chronic disease and the widely observed phenomenon of increased production of inflammatory mediators that accompany this process, referred to as “inflammaging.” While it has been demonstrated that the gut microbiome and immune system interact, the relationship between the gut microbiome and age remains to be clearly defined, particularly in the context of inflammation. The aim of our study was to clarify the associations between age, the gut microbiome, and pro-inflammatory marker serum MCP-1 in a C57BL/6 murine model.

**Results.** We used 16S rRNA gene sequencing to profile the composition of fecal microbiota associated with young and aged mice. Our analysis identified an association between microbiome structure and mouse age and revealed specific groups of taxa whose abundances stratify young and aged mice. This includes the Ruminococcaceae, Clostridiaceae, and Enterobacteriaceae. We also profiled pro-inflammatory serum MCP-1 levels of each mouse and found that aged mice exhibited elevated serum MCP-1, a phenotype consistent with inflammaging. Robust correlation tests identified several taxa whose abundance in the microbiome associates with serum MCP-1 status, indicating that they may interact with the mouse immune system. We find that taxonomically similar organisms can exhibit differing, even opposite, patterns of association with the host immune system. We also find that many of the OTUs that associate with serum MCP-1 stratify individuals by age.

**Discussion.** Our results demonstrate that gut microbiome composition is associated with age and the pro-inflammatory marker, serum MCP-1. The correlation between age, relative abundance of specific taxa in the gut microbiome, and serum MCP-1 status in mice indicates that the gut microbiome may play a modulating role in age-related inflammatory processes. These findings warrant further investigation of taxa associated with the inflammaging phenotype and the role of gut microbiome in the health status and immune function of aged individuals.

## Introduction

Aging is accompanied by a progressive decline of several physiological functions, predisposing the host to impaired function and increased mortality risk (López-Otín et al., 2013). Age is the primary risk factor for major human chronic diseases, including cancer, type 2 diabetes, cardiovascular disorders, and neurodegenerative diseases. The immune system is particularly sensitive to age-related alterations in function. Aging of the immune system, or immunosenescence, contributes to increased susceptibility to infection, autoimmune diseases, chronic inflammatory diseases, and cancer. Immunosenescence encompasses both the impairment and dysfunction of adaptive and innate immune responses (Franceschi et al., 2007; Gruver, Hudson & Sempowski, 2007; Provinciali et al., 2010). Age-dependent dysregulation of immunity may play an important role in aging and the widely observed phenomenon of increased production of inflammatory mediators that accompany this process, referred to as “inflammaging” (Franceschi, 2007; Shaw, Goldstein & Montgomery, 2013). However, there is wide variability in the overall inflammatory response of age-associated basal inflammation across populations (Shaw, Goldstein & Montgomery, 2013). We currently do not have a complete understanding of the mechanisms that produce variability in inflammatory mediator production associated with aging or factors that may explain variable susceptibility to this process among individuals (Chung et al., 2009; Cevenini et al., 2010).

One factor, the host gut microbiome, has been suggested to be an important determinant of human susceptibility to several age-related conditions, including metabolic syndrome and cancer (Cho & Blaser, 2012; Claesson et al., 2012). There is accumulating evidence that aging may be associated with changes in the gut microbiome in invertebrates, such as *Caenorhabditis elegans*, and vertebrates, including rodents and humans (Biagi et al., 2010; Rampelli et al., 2013; Heintz & Mair, 2014; Langille et al., 2014). Of growing interest is the relationship between aging and gut microbiome diversity. Most studies have focused on the relatively rapid diversification of the gut microbiome that occurs during early human development (i.e., infancy to three years of age) (Hopkins, Sharp & Macfarlane, 2002; Koenig et al., 2011). However, a limited number of studies have described a human lifespan-associated trend in microbiome diversification, a pattern that appears to be consistent across distinct human populations (Mariat et al., 2009; Yatsunenko et al., 2012). Given that changes in microbiome composition can associate with chronic disease (Ley et al., 2006; Turnbaugh et al., 2006; Ley, 2010; Murphy et al., 2010; Koeth et al., 2013; Gregory et al., 2015; Chassaing et al., 2015) and that specific microbiota can interact with the immune system to regulate inflammation (Atarashi et al., 2011; Round et al., 2011), it has been hypothesized that age-related changes in the microbiome associate with and potentially contribute to the proinflammatory environment associated with the aging process (Magrone & Jirillo, 2013; Heintz & Mair, 2014). However, to date there is limited data available regarding alterations in the gut microbiome with age and its relationship to inflammation.

Mouse models provide a controlled setting in which specific interactions between hosts and their microbiome can be empirically explored. The use of mouse models has clarified specific associations between mammalian physiology and the microbiome and identified causal mechanisms employed by the microbiome to modulate host physiology and vice versa (Hooper et al., 2001; Geuking et al., 2011; Vaishnava et al., 2011). However, their application to the study of the interaction between aging and the microbiome has been limited. Two prior studies have used mouse models to investigate this interaction and identified age-related differences in the mouse gut microbiome (Murphy et al., 2010; Langille et al., 2014), but it is generally unclear how these differences correspond to age-related immunological variation. Characterizing this relationship is useful given that mouse models of aging elicit immunological profiles consistent with age-related inflammation in humans (Wong & Ho, 2012) and that accumulating evidence suggests that microbiome structure can modulate the mammalian innate and adaptive immune system by regulating a delicate balance of pro- and anti-inflammatory responses (Magrone & Jirillo, 2013; Chu & Mazmanian, 2013).

Age-related inflammation may be a major contributor to several age-related disorders. The identification of mechanisms contributing to age-related inflammation could have a significant impact on improving the quality of life for older individuals. Pro-inflammatory chemokines and cytokines, typified by monocyte chemoattractant protein-1 (MCP-1), may serve as biomarkers of inflammatory processes that underlie aging and age-related diseases (Conti & DiGioacchino, 2001; Deshmane et al., 2009). MCP-1/CCL2 is a 76–amino-acid peptide that serves as the major lymphocyte chemoattractant secreted by mitogen-stimulated peripheral blood mononuclear cells (Deshmane et al., 2009). MCP-1 was first shown to be positively associated with age in a study of 405 healthy Japanese subjects (Inadera et al., 1999) and has since been confirmed in animal models and other human studies (Tyagi et al., 2014). Here, we explore the relationship between age, the microbiome, and serum MCP-1 as a surrogate marker of inflammation in a mouse model.

## Materials and Methods

### Animals, diets, and sample collection

Young (2 mo.) and aged (26 mo.) female C57Bl/6 mice were purchased from the aged rodent colonies at the National Institute on Aging (Bethesda, MD). All mice were healthy and free from obvious signs of illnesses and tumors. Five mice from the same age group were co-housed in a temperature- and humidity-controlled environment and were fed an AIN93 diet (Wong et al., 2009). Diets were purchased from Research Diets (New Brunswick, NJ). Mice were maintained on the purified diets for a total of five weeks. Food and water were provided *ad libitum*. Dietary intakes and body weights of all mice were monitored throughout the entire study. Fecal samples were collected after four weeks and stored at −20 °C. Blood samples were also collected, and serum samples were immediately frozen after separation and stored at −80 °C. Mice were euthanized by CO_2_ asphyxiation at the termination of the experiments. The animal protocol was approved by the Oregon State University Institutional Laboratory Animal Care and Use Committee under ACUP 4204.

### Pro-inflammatory cytokine measurements

Profiles of the pro-inflammatory cytokine MCP-1 in serum were determined using a BD cytometric bead array mouse inflammation kit (BD Biosciences, San Jose, CA, USA). Quantitative measurements of MCP-1 were determined by flow cytometry. Data were acquired using FACSCalibur (BD Biosciences), and data analyses were conducted using FCAP Array Software version 3.0 (BD Biosciences).

### Fecal DNA isolation and 16S amplicon sequencing

Fecal DNA was isolated using QIAamp DNA stool mini-kits (Qiagen, Valencia, CA, USA) per manufacturer’s instructions. 16S rRNA PCR amplification was conducted according to established methods (Caporaso et al., 2012). Briefly, each sample’s extracted DNA was subjected to polymerase chain reactions to amplify the V4 region of the 16S locus using PCR primers (515F and 806R) that include Illumina adapters and sample-specific barcodes. PCR amplicons from individual mouse samples were cleaned using the QIAquick PCR cleanup kit (Qiagen) and pooled. An aliquot of the pooled 16S library was sequenced on an Illumina MiSeq (v3 chemistry) at the Center for Genome Research and Biocomputing core facility (Oregon State University, OR). This generated ∼3.99 million 300bp single end reads (median reads per sample = 395,310).

### Bioinformatic and statistical data analysis

The QIIME software package (v1.8.0) was applied using default parameters to quality control sequence data and to quantify the diversity of microbial communities, as described previously (Rideout et al., 2014). Specifically, the QIIME script split_libraries.py was used with default parameters to trim and filter low quality sequences (i.e., quality < 25) and remove reverse primers. Operational Taxonomic Units (OTUs) were identified using open-reference OTU picking in QIIME via the pick_open_reference_otus.py script, using the UCLUST (v1.2.22) algorithm against the Greengenes 97% OTU reference database (v13.8) (Kopylova et al., 2016). OTUs were phylotyped using the assign_taxonomy.py script in QIIME, using UCLUST (v1.2.22) as an assignment method and the GreenGenes 97% OTU database (v13.8) as an annotation reference. Samples were rarefied to 200,000 reads, and alpha- (i.e., richness) and beta-diversity (i.e., weighted and unweighted UniFrac distances) were subsequently quantified using the core_diversity_analyses.py script in QIIME.

Statistical analyses were conducted in R. The coin package was used to implement robust statistical tests and identify differences in the gut microbiome communities of young and aged mice (i.e., Wilcoxon tests), and the Kendall package was used to quantify the correlation between serum MCP-1 and OTU abundance (i.e., Kendall’s tau). Linear models relating OTU abundance and MCP-1 were constructed using the lm function. For the co-variation analysis, OTU’s were filtered based on presence in ≥50% of samples. False discovery rates were quantified using the q-value software package (Storey, Taylor & Siegmund, 2004), except for phylum level analyses, in which Bonferroni corrections were conducted using the p.adjust function in R, given the small number of tests.

## Results

### Mouse fecal microbiota composition varies by age

**Figure 1:**
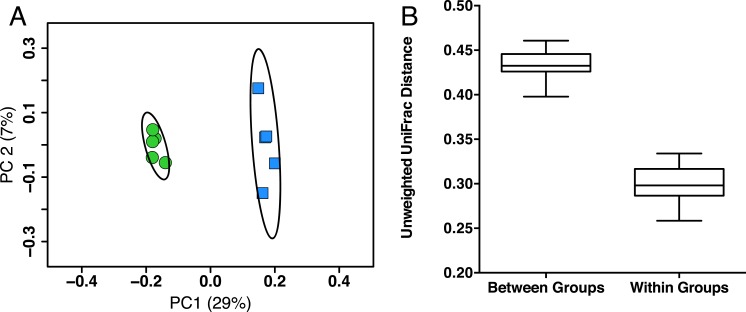
Young and aged mice are distinctive at the beta-diversity level using UniFrac metric. (A) Principal coordinates analysis using unweighted UniFrac distance on 16S sequences from fecal microbiota of young (2 months, blue) and aged (26 months, green) mice showing there are distinct phylogenetic differences in the gut microbiome between young and aged mice. Ellipses represent 95% confidence intervals. (B) Within-age-group beta-diversity is significantly lower than the between-age-group diversity, indicating that the composition of gut microbiomes from aged mice significantly differs from those of young mice (taxon abundance weighted and unweighted UniFrac; p < 0.01, Bonferroni corrected non-parametric t-tests’).

In order to identify possible gut microbiota signatures with age, we compared the gut microbiota composition of five young mice (two months old, female, C57Bl/6) to five aged mice (26 months old, female, C57Bl/6). We first assessed the intersample diversity between the young and aged mice. We used UniFrac, which normalizes intersample taxonomic differences by the phylogenetic diversity of the microbial lineages observed in the samples (i.e., samples containing more phylogenetically similar taxa produce a relatively lower distance). A principal coordinates analysis (PCoA) of fecal samples based on their unweighted UniFrac distances reveals that samples primarily cluster by age, which suggests that the young and aged mice exhibit microbial communities with different evolutionary histories (Fig. 1A). Non-parametric tests support this observation, as the intra-age UniFrac distances are significantly smaller (Bonferroni-corrected Wilcoxon test *p* < 0.01) than the inter-age distances (Fig. 1B). These results are qualitatively consistent with those obtained using weighted UniFrac and indicate that there are distinct phylogenetic differences in the gut microbiome between young and aged mice.

**Figure 2:**
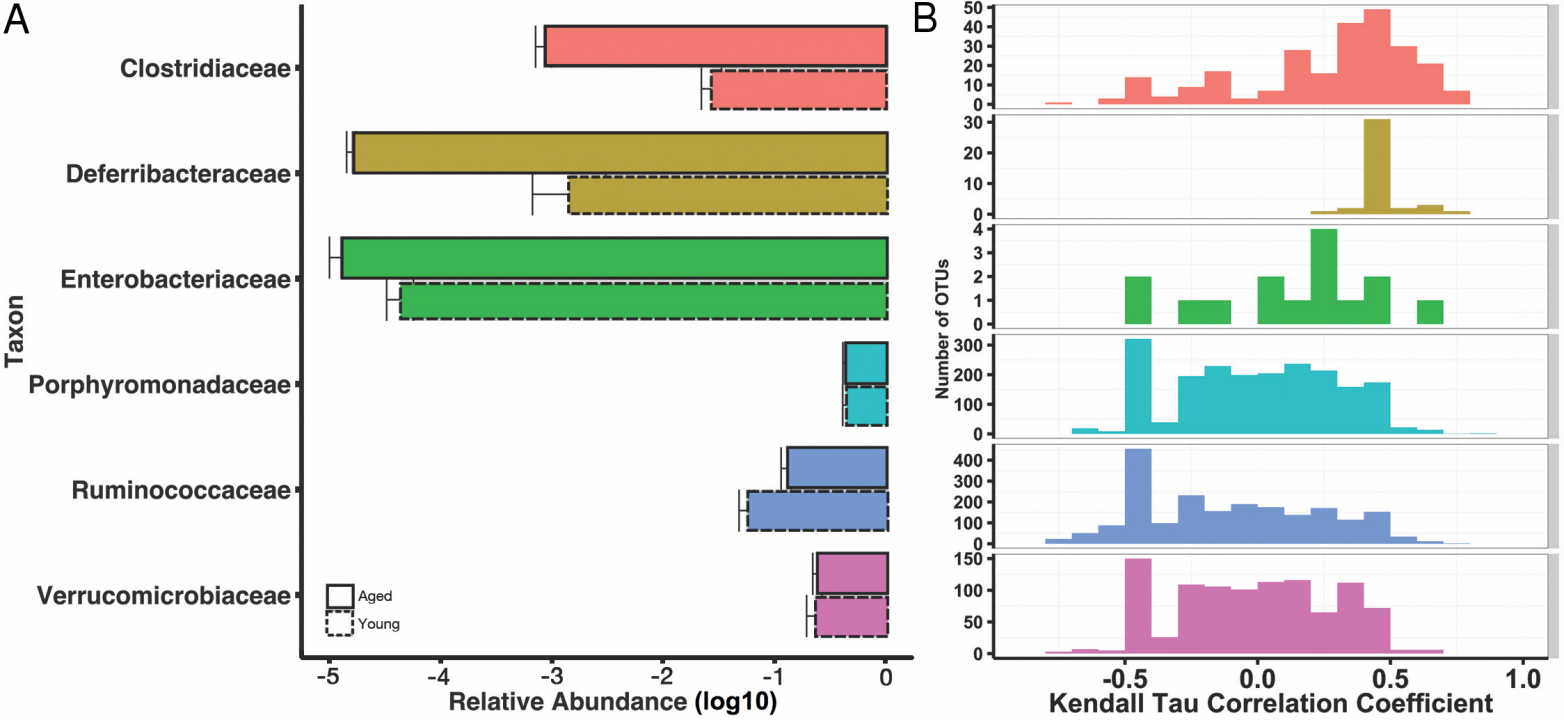
Some age-associated differences in mouse gut microbiome also correlate with inflammatory marker MCP-1. (A) Selected significant taxonomy representing differences between young (dotted line) and aged (solid line) at the family level, expressed as mean relative abundances on a log10 scale. (B) Histograms for Kendall tau correlation coefficients representing the complex relationship between individual OTUs and serum MCP-1 by taxonomic family. Certain family level differences observed between young and aged mice also appear to correlate with pro-inflammatory marker, serum MCP-1.

We then explored the structure of these communities at various taxonomic levels to understand potential taxonomic signatures that may be driving the observed age effect. Using 16S rRNA ribosomal genes as a marker, sequences were clustered into operational taxonomic units (OTUs) using a threshold of 97% sequence similarity from the GreenGenes database with the QIIME open-reference OTU-picking protocol. OTUs were then taxonomically annotated using UCLUST. As expected, given prior characterizations of the mouse gut microbiome (Nguyen et al., 2015), all mice were dominated by the phyla Bacteroidetes, Firmicutes, and Verrucomicrobia, and these phyla did not stratify young and aged mice. We found increased abundances of Deferribacteres and Bacteroidetes in the aged mice both marginally significant (*p* = 0.055, *p* = 0.11, respectively). At the level of families, Ruminococcaceae and Christensenellaceae were overrepresented in the young mice (*q* < 0.15), whereas Clostridiaceae and Enterobacteriaceae were more abundant in the aged (*q* < 0.15). The genera *Oscillospira* and *Blautia* increased in the young mice, whereas *Mucispirillum, Eggerthella, Clostridium, Sarcina*, and *Anaerotruncus* were increased in the aged mice (*q* < 0.15). These results are summarized at the family level in Fig. 2A, and a complete table of the taxa that stratify mice by age, as well fold change between groups, can be found in (Table S2).

### Specific gut microbiota are associated with pro-inflammatory marker serum MCP-1

**Figure 3:**
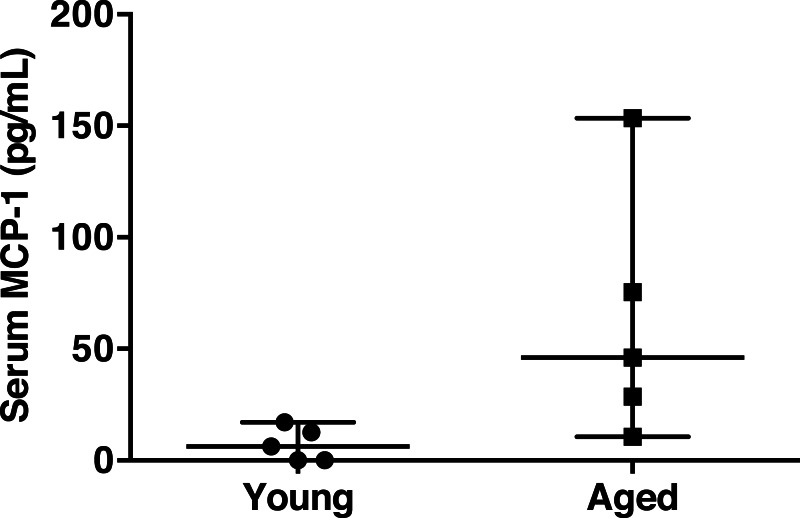
Serum MCP-1 distribution of aged and young mice. Mean serum MCP-1 levels in aged mice are significantly higher than in young mice (62.8 ± 25.0 versus 7.2 ± 3.4 pg/mL, respectively, Wilcoxon-test p = 0.036). Points represent individual mice. Lines represent minimum, median, and maximum values.

Chronic, low-grade, systemic inflammation is associated with aging and contributes to immunosenescence (Shaw, Goldstein & Montgomery, 2013). To test the hypothesis that specific gut microbiota are associated with an increase in pro-inflammatory marker serum MCP-1, we correlated taxon abundances with serum MCP-1, since it was strongly associated with aging. Circulating MCP-1 was significantly elevated in the aged mice compared to young mice (62.8 ± 25.0 versus 7.2 ± 3.4 pg/mL, respectively, Wilcoxon-test *p* = 0.036) (Fig. 3). However, serum MCP-1 was highly variable in the aged animals. This observation of inconsistent levels of MCP-1 among the aged mice is comparable to the variation in specific inflammatory mediators observed in aged humans (Shaw, Goldstein & Montgomery, 2013), wherein chronic inflammation develops with age, but the hallmarks and degree of inflammation are quite varied.

**Figure 4:**
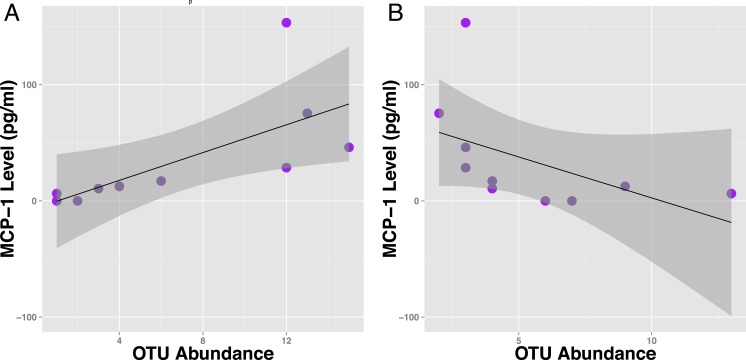
Specific OTUs are correlated with serum MCP-1 status in mice. Example of two OTUs from within the same genus (Parabacteroides) showing positive and negative correlations of OTU abundance to MCP-1 status, suggesting OTUs from within same order are inconsistent with correlations with MCP-1. Plot A shows OTU12369 (tau =.78) and Plot B shows OTU71089 (tau = − .66). Points represent individual mice. Black lines represent a linear model of the data with standard errors represented by the surrounding dark grey shaded area. OTU abundance represents the number of sequences classified into an OTU with 97% sequence similarity after they were rarified by a sequencing depth of 200,000.

If a specific taxon interacts with the immune system, we might expect its relative abundance in the microbiome to associate with cytokine abundance. We tested for such associations by using Kendall’s tau to quantify the correlation between each OTU’s abundance and MCP-1. We identified 293 OTUs that significantly (*q* < 0.15, tau > 0.5) associate with MCP-1 status (Fig. 2B). Of these, 117 OTUs positively associate with MCP-1, including OTUs within *Parabacteroides* (tau= 0.84), *Mucispirillum* (tau = 0.69), *Clostridium* (tau = 0.69), and *Sarcina* (tau = 0.69), which show the strongest correlations. Conversely, 176 OTUs negatively correlate with MCP-1. Those with the strongest negative correlations are within *Akkermansia* (tau = − 0.75), *Oscillospira* (tau = − 0.78), *Blautia* (tau = − 0.76), and *Lactobacillus* (tau = − 0.75). Among families, the Clostridiaceae contained the largest percentage of OTUs that significantly correlate with MCP-1 (22%), followed by Ruminococcaceae (11%). Many genera and families contain multiple OTUs that significantly associate with MCP-1 (Fig. 2). However, these associations are not always consistent among the OTUs that comprise a taxonomic group. For example, the Porphyromonadaceae family contains OTUs that both positively and negatively correlate with MCP-1, indicating that even closely related microorganisms may exhibit diverse or inconsistent interactions with the host (Fig. 4). Many of the OTUs with the strongest positive and negative associations are uncharacterized at the family and genus level, especially among the order Clostridiales. A complete list of these results can be found in (Table S1).

## Discussion

Emerging evidence indicates that the human gut microbiome diversifies in an age-related manner (Mariat et al., 2009; Yatsunenko et al., 2012; Langille et al., 2014). It is currently unclear how this diversification relates to the development of age-associated chronic diseases. While human studies provide clinically relevant insight, they introduce many external variables that are difficult to control. Mouse models are valuable because they enable controlled experimentation, and for this reason they have been used to study the interaction between the gut microbiome and host physiology or health status, including inflammatory bowel disease, metabolic syndrome, and obesity (Turnbaugh et al., 2006; Manichanh et al., 2012; Kostic, Xavier & Gevers, 2014; Chassaing et al., 2015). Currently, our understanding of lifespan-related changes in the composition of the enteric microbiome in mice is limited to two studies. Zhang et al. (2013) describe age-related gut microbial shifts in mice on a calorie-restricted diet from 5 to 141 weeks of age, while Langille et al., (2014) recently characterized microbial compositions in young, middle-aged, and old mice in relation to the development of frailty. Both studies identified age-related differences in the gut microbiome but differed in how specific taxa associate with age. Additionally, aside from Langille et al.’s exploration of the phenotype of host frailty, there is almost no insight into how age-associated aspects of the gut microbiome correspond to mouse physiology, such as age-related inflammation.

The goal of this study was to clarify the relationship between gut microbiota composition, age, and MCP-1 in mice. We investigated the composition of gut microbiome communities in five young mice and five aged mice, profiled their serum marker MCP-1 levels, and correlated our observations to identify potential interactions between specific microbiota and serum MCP-1. We observed the young and aged groups of mice had distinct gut microbiomes at the level of beta-diversity and taxonomic structure. We also found that aged mice exhibit elevated serum MCP-1. Further, some of the taxonomic differences in the microbiome observed between young and aged mice are strongly correlated with serum MCP-1 status. This suggests that differences in the gut microbiome observed between young and aged groups of mice may be associated with the age-related development of increased pro-inflammatory marker MCP-1, but causal relationships have not yet been explored.

We compared our results with the other two available age-related mouse microbiome investigations to evaluate whether the mouse gut microbiome consistently diversifies with age. Our data are consistent with previous reports that the mouse gut microbiome is largely dominated by the phyla Bacteroidetes and Firmicutes (Nguyen et al., 2015). We did not observe any notable differences in the relative ratio of Firmicutes to Bacteroidetes between age groups. This observation is consistent with the patterns identified in Langille et al. (2014) and differs from Zhang et al.’s (2013) finding of a large shift from Firmicutes to Bacteroidetes in the microbiomes of calorically restricted aged mice.

In our study, the phylum Deferribacteres exhibited marginally significant differences in abundance between the young and aged mice (*p* = 0.055, Bonferroni corrected non-parametric *t*-test). These differences are entirely driven by variation in the genus *Mucispirillum* (*p* = 0.008), which has a two orders of magnitude difference in mean abundance between the young and aged mice. While *Mucispirillum*, a mucin-degrading bacterium (Robertson et al., 2005), is not a well-understood component of the human gut microbiome, it appears to be important in mouse models of inflammation. For example, its relative abundance in the gut is elevated in two different mouse models of colitis compared to controls (Rooks et al., 2014; Belzer et al., 2014). Increased abundance of *Mucispirillum* also coincides the transient pro-inflammatory response observed upon colonization of germ-free mice (El Aidy et al., 2014). We also found that 50% of the *Mucispirillum* OTUs identified in our investigation are positively correlated with MCP-1 (*q* < 0.15). Prior work suggests *Mucispirillum* can induce inflammation, possibly through mucin degradation. Reduction of the mucus layer may allow for potentially greater access of luminal antigens to the gut immune system and activation of the inflammatory response (Ganesh et al., 2013). Follow-up experimentation can determine if *Mucispirillum* plays a role in the development of age-related inflammation.

Most of the taxonomic differences between young and aged mice were observed at the family and genus levels. Among the families, the Ruminococcaceae were overrepresented in the young mice, while Clostridiaceae and Enterobacteriaceae were more abundant in the aged mice. In addition to *Mucispirillum*, the genera *Eggerthella*, *Clostridium*, *Sarcina*, and *Anaerotruncus* were significantly more abundant in the aged mice. Some of these observations coincide with Langille et al.’s (2014) work, which found similar differences in the Ruminococcaceae and Clostridiaceae, while others diverge, including their unique finding of age-related differences in the Lachnospiraceae and our observation of variation in the Enterobacteriaceae. These inconsistent results indicate that some mouse gut microbiota robustly associate with age, while others may be subject to study-specific or institutional variation.

Our study is the first to evaluate the relationship between an immunological marker that has been shown to associate with age-related inflammation, MCP-1 (Conti & DiGioacchino, 2001; Deshmane et al., 2009; Mansfield et al., 2012), and the mouse gut microbiome. In our study, aged mice exhibited significantly elevated serum MCP-1 relative to young mice. However, there was large variation in serum MCP-1 status among aged individuals. We correlated serum MCP-1 status with OTU abundance and found that many of the OTUs that associate with this marker of inflammation are members of families that also are associated with gut microbiome differences observed between young and aged groups of mice. These results indicate that some of the differences in the gut microbiome that associate with age also associate with MCP-1. An expanded evaluation with a panel of pro-inflammatory cytokines is needed to clarify if these changes promote inflammatory processes in aging. Since not all aged mice in this study developed the elevated MCP-1 phenotype, which is consistent with human populations, it is tempting to speculate that gut microbiome composition may contribute to the development of inflammaging.

We found 120 OTUs that positively correlated with serum MCP-1. One of the families that most consistently associated with MCP-1 is the Clostridiaceae. Microorganisms within this family have been shown to positively associate with mucosal inflammation and are directly correlated with mucosal ulceration (Scarpa et al., 2011; Jiang et al., 2015). The Clostridiaceae are also significantly increased in our aged mice. Conversely, 162 OTUs negatively correlated with MCP-1. These taxa may induce an anti-inflammatory response or may be especially sensitive to inflammation. Over 40% of these OTUs are found in the Ruminococcaceae family, which was also relatively elevated in young mice. The Ruminococcaceae have been described as part of a core healthy gut microbiome, and decreases in abundance within this family have been observed across the human lifespan and are also associated with colonic inflammation (Hayashi et al., 2003; Li, Bihan & Methé, 2013; Perez-Muñoz et al., 2014). Over 15% of the OTUs that negatively correlate with MCP-1 were taxonomically annotated to the genus *Oscillospira*, which is a member of the Ruminococcaceae family that demonstrated the largest difference in abundance between young and aged groups of mice. Prior work has identified a negative correlation between *Oscillospira* abundance and inflammatory pathways that regulate barrier function in the colon (Hamilton et al., 2015). Collectively, these results support the notion that different taxonomic groups of microbiota can differentially correlate with the host immune system. Further, many of the taxa that stratify the young and age mice, such as *Eggerthella*, *Sarcina*, and *Anaerotruncus* also appear to be associated with inflammation in prior investigations (Thota et al., 2011; Candela et al., 2014). This suggests the gut microbiome may play a role in the development of age-associated inflammation.

However, our work also reveals that the patterns of interaction between OTUs within a taxonomic group and their host are complex and should be interpreted with caution. Plotting OTU and MCP-1 correlation coefficients across families reveals a broad coefficient distribution (Fig. 2). For example, of the six families that contain more than one OTU that significantly associates with MCP-1, five of them exhibit coefficient distributions that span both positive and negative coefficients. While associations within families are complex, some families exhibit biases towards positive (i.e., Clostridiaceae) or negative (i.e., Ruminococcaceae) coefficients. This indicates that for some families, general patterns of host-microbe interaction may exist, at least for serum MCP-1. We find that one individual appears to be highly elevated in its inflamed status relative to the rest of the aged cohort. We verified that the results discussed here are robust to whether this outlier is included in our correlation analyses. Ultimately, further experimentation is required to validate the functional roles of these taxa and their interaction with their host.

Our work has implications for future studies examining the relationship of the gut microbiome and the immune system in the context of age. Diversification of the gut microbiome in association with age may contribute to age-related inflammation through mediation of nutrient metabolism and immunity (Rehman, 2012; Magrone & Jirillo, 2013). This may include differences in bacterial metabolites and production of inflammatory mediators influencing local and systemic processes, such as gut permeability and immune function. This may, in turn, increase the host’s susceptibility to inflammation and related chronic diseases (Joyce & Gahan, 2014). Given the immune system’s ability to sense commensal microbiota and trigger various immune-related responses (Macpherson & Harris, 2004; Hooper, Littman & Macpherson, 2012), we speculate that taxa with strong positive or negative correlations to MCP-1 could be directly or indirectly interacting with the immune system. Our work supports the hypothesis that age-related diversification of the gut microbiome contributes to the immunological variation that is also observed with age. In consideration of work that shows immune-compromised hosts have altered gut microbial composition (Garrett et al., 2007), it is likely the immune system is also affecting microbial composition.

While our experiment has clarified how the mouse gut microbiome varies in association with age and has yielded hypotheses about how microbiota interact with the immune system, it is limited by several factors. Similar to other studies of gut microbiome and aging in mice, our analyses are confounded by potential cage effects, a small number of animals, and the use of a single marker of inflammation. Investigations of aging in mice, including the previous studies of the gut microbiome and age, also commonly use individuals from different cohorts to obtain time-matched individuals at different points in their lifespan (Sprott, 1991). This is frequently necessary given the costs associated with maintaining laboratory animals over the entirety of their lifespan. Our study adopted a consistent design, and as a result, it is difficult to account for potential cohort effects. However, the strong overlap between our study and other investigations suggests that many of the findings reported here are robust to these study effects. MCP-1 was used a surrogate marker of inflammation and oversimplifies the potential relationship associating inflammatory processes associated with aging. Confirming age-related associations for other immunological markers will help establish important bi-directional pathophysiologic mediators for the aging process. Furthermore, MCP-1 abundance may not reflect the results of aging but may be the result of inflammatory changes wrought by atherosclerosis, endotoxemia, or other covariates of age-related, chronic inflammatory conditions. Since we did not measure other biomarkers of these potential confounders or covariates, we cannot rule out other potential explanations for these interactions (Cani et al., 2008; Bennett et al., 2013; Koeth et al., 2013; Gregory et al., 2015). Regardless, our work provides important insight into the potential interactions between host age, inflammation, and the gut microbiome and improves the contextualization of the age-associated diversification of the mouse gut microbiome observed in the limited number of prior studies.

## Conclusions

Recent work suggests that the gut microbiome diversifies in association with host age. Here, we detected age-related differences in the gut microbiome that are associated with the serum marker MCP-1 in a murine model. The correlation between age, relative abundance of specific taxa in the gut microbiome, and serum MCP-1 status in mice indicates that the gut microbiome may play a modulating role in age-related immunological processes. These findings warrant further investigation of taxa associated with the inflammaging phenotype and the role of gut microbiome in the health status and immune function of aged individuals.

## Supplemental Information

10.7717/peerj.1854/supp-1 Table S1 OTU abundance and serum MCP-1 correlation data

10.7717/peerj.1854/supp-2 Table S2 Taxonomic differences observed between young and aged mice

## Additional Information and Declarations

### Data Availability

The following information was supplied regarding data availability: BioProject ID PRJNA311095, accessions SRX1581953, SRX1581958– SRX1581960, and SRX1571787– SRX1571792

http://files.cgrb.oregonstate.edu/Sharpton_Lab/Papers/Conley_PeerJ_2015/.
